# Cervical cancer screening and HPV genotype distribution among asymptomatic patients of Karachi Pakistan

**DOI:** 10.12669/pjms.313.8004

**Published:** 2015

**Authors:** Mubeena Shahid, Shahana Urooj Kazmi, Ameena Rehman, Jahanara Ainuddin, Sayed Furqan, Sobia Nazeer

**Affiliations:** 1 Mubeena Shahid, MBBS, M-Phil Immunology and Infectious Disease Research Laboratory, Department of Microbiology, University of Karachi, Karachi - Pakistan; 2 Prof. Dr. Shahana Urooj Kazmi, PhD University of Karachi, Pakistan; 3 Ameena Rehman, FCPS Civil Hospital Karachi, Pakistan; 4 Jahanara Ainuddin, FCPS, PhD Civil Hospital Karachi, Pakistan; 5 Sayed Furqan, MCPS National Institute of Child Health, Karachi, Pakistan; 6 Sobia Nazeer, Medical Technologist, Liaquat National Hospital, Karachi, Pakistan

**Keywords:** Asymptomatic, Cervix cancer, HPV genotypes, Socio-demographic factors

## Abstract

**Background:**

Cervical cancer is the second most prevalent cancer in females worldwide. Human papillomavirus (HPV) infection is a sexually transmitted infection. However, in addition to HPV infection, other factors exist that influence the risk of developing cervical cancer. In Pakistan most women who developed cervical cancer have been infrequently or never screened.

**Objective:**

To determine the prevalence of HPV infection and its subtype profile among asymptomatic patients with pre cancerous cervical intraepithelial lesion.

**Methods:**

In this hospital-based descriptive study, 160 asymptomatic females attending gynecology clinics were subjected to HPV screening after obtaining informed consent. Cervical Scrapings were examined by cytopathology and colposcopic directed biopsies taken. High-grade intraepithelial lesion (HSIL) CIN-2, and Low-grade intraepithelial lesion (LSIL) CIN-1 were selected. Samples were analyzed for the presence of HPV-DNA general and type specific genotype 16 and 18. HPV- DNA was extracted by QIA amp DNA kit protocol and amplification was done by polymerase chain reaction (PCR) and genotyped by type specific primers.

**Results:**

Out of 160, 17 Pap smear tests were positive, 6 (35.3%) with abnormal results (HSIL) CIN-2 were HPV-DNA positive. Among them, 5 (83.3%) had subtype 16 and in 1 (16.7%) case the genotype was undetectable. The remaining 11(6.9%) with pre cancer minimal abnormal (LSIL) CIN-1 presented. Out of them 3 (27.3%) were HPV-DNA positive with subtype 16. Five (45.4%) were followed by repeated pap smear every six months for two years, and the rest of 3 (27.3%) patients refused for the test.

**Conclusion:**

A high incidence of Human papillomavirus (HPV) infection is found in women with pre cancerous lesion of cervix in Pakistani women.

## INTRODUCTION

It is now well established that HPV infection is the central, probably necessary cause of cervical cancer.^1^ Statistical analyses released from the World Health Organization (WHO) suggest that cervical cancer is the second most common cancer in women worldwide.^2^,^3^ It is estimated that each year approximately 493,000 new cases are diagnosed and 274,000 women die from cervical cancer worldwide.^4^ The incidence of cervical carcinoma is substantially higher among women of low socioeconomic status. According to a WHO study, the incidence of cervical cancer in Pakistan in 2008 was 13.6 per 100,000 compared to less than 9 per 100,000 in 2002, which shows that the country is moving from low-risk to moderate risk level, making it a danger zone where young girls are more at risk than before. Although Pap smear screening has decreased the incidence of cervical cancer in the United States, there are still pockets of the population, such as the American Indian women of the Northern Plains, that have a significantly higher rate of cervical cancer.^5^,^6^

Human papillomavirus is considered to be the most important risk factor in the development of cervical cancer and sexual transmission is the predominant route of HPV infection. Transitional zone of cervix is the most common site of cervical cancer and it is most susceptible to the carcinogenicity of HPV.^7^

The cofactors may be genetic, immunological as well as sociodemographic, e.g. lower age of conception, high parity, use of oral contraceptives, diet, smoking, etc. it was also evidenced that women co-infected with multiple HPV-type infections comprising of one or more high-risk types were prone to persistent HPV infection.^8^

General improvement in socioeconomic status and educational level of the population tends to have a positive effect on the risk of cervical cancer by altering some of the known risk factors such as age at marriage, parity and health-care seeking behavior. Other strategies such as low-intensity cytology screening (e.g., 1 Pap smear every 10 years after age 35) and visual inspection need to be better evaluated in randomized controlled trials to determine their cost-effectiveness.^9^

Vaccination against HPV may have greatest value in developing countries, where 80% of the global burden of cervical cancer occurs each year and where Pap screening programs have been largely ineffective. Two main types of vaccine are currently being developed: prophylactic vaccines to prevent HPV infection and consequently the various HPV-associated diseases, and therapeutic vaccines to induce regression of precancerous lesions or remission of advanced cervical cancer.

The study was done to determine the prevalence of HPV infection and its subtype profile among asymptomatic Pakistani women with pre cancerous cervical intraepithelial lesion. Details of Socio-demographic variables were also studied.

## METHODS

### Study design

This is descriptive study. Taking the incidence of HPV infection in cervical cancer as 65.8%^10^ with 7% confidence limit, at 95% confidence level, sample size calculated was 177.

### Patient selection

Women attending OPD of tertiary care hospital for routine physical gynaecological examination from April 2012 to December 2012 in Karachi participated in this study. An appropriate institutional review board approval was obtained prior to sample collection. Written informed consent was obtained from 160 voluntary patients to perform a conventional Pap smear test. Cervical sample was prepared on a glass slide immediately sprayed with fixative 95% alcohol for later testing. All Positive (HSIL/LSIL) patients were re- examined by expert Gynecologist using colposcope and cervical scraping exfoliated cervical cells in tubes of 5ml pre-chilled phosphate buffered saline (PBS) and colposcopic directed biopsy was taken from suspicious areas. The pap smears were examined by expert Cytologist/Histopathologist using the Bethesda classification. Colposcopy and histology results were reported as per the Richart CIN system in which the terms atypical squamous cells of undetermined significance (ASCUS), squamous intraepithelial lesion (LSIL, HSIL) are used.^11^-^13^

### DNA extraction of cervical cells and tissue biopsies

Biopsy tissue crushed and re-suspended in chilled phosphate buffered saline (PBS) then DNA was extracted by QIA amp DNA Mini Kit as per Manufacturers instructions (Qiagen QIA amp kit) Germany.

### Polymerase chain reaction

The samples were analyzed by polymerase chain reaction (PCR), using four sets of primers, that is, GP5/GP6, general primers for HPV, TS16-A/TS16-B and TS18-A/TS18-B, subtype-specific primers for HPV subtypes 16 and 18 respectively, and C03/PC04 for b-globin, sequences for the primers are given in Table-I along with the length of respective PCR amplimers and the targeted gene. The PCR conditions for these primers were as follows.

**Table-I:** Primers used for HPV typing.

Primer	Sequence	Target gene	Amplimer length
GP5	TTTGTTACTGTGGTAGATAC	L1	155 base pair
GP6	TAAAAATAAACTGTAAATCA		
TS16-A	GGTCGGTGGACCGGTCGATG	L1	96 base pairs
TS16-B	GCAATGTAGGTGTATCTCCA		
TS18-A	CCTTGGTAAATTTTTGG	L1	115 base pairs
TS18-B	CACGCACACGCTTGGCAGGT		
PCO3	ACACAACTGTGTTCACTAGC	Β-globin	110 base pairs
PCO4	CAACTTCATCCACGTTCACC		

Reference for each primer are cited in the materials and methods section. HPV, human papillomavirus, GP, general primer for L1 HPV, TS, type specific primers for L1 HPV, PCO primers for β-globin gene.

**Table-II:** Demographic features of asymptomatic women participants (N=160).

Parameters		Pap Smear +ve 17 (10.62)^Y^		Pap Smear –ve 143 (89.37)^Y^	
		No.	%	No.	%
Age (years)	≤40	9	15.5	49	84.5
	>40	8	7.8	94	92.2
Marital Status	Married with one wife	10	7.2	129	92.8
	Married with >1 wife	7	33.3	14	66.7
Age at sexual (years)	≤20	7	20.6	27	79.4
	>20	10	7.9	116	92.1
Education	Illiterate	12	31.6	26	68.4
	Literate	5	4.1	117	95.9
Residence	Urban	7	6.2	106	93.8
	Rural	10	21.3	37	78.7
Income	Low	9	17	44	83
	Middle	8	7.5	99	92.5

**Table-III:** Risk factors associated with women who are pap smear positive (N=160).

Parameters		Pap Smear +ve 17 (10.62)^Y^		Pap Smear –ve 143 (89.37)^Y^	
		No.	%	No.	%
Post Coital Bleeding	Yes	12	54.5	10	45.5
	No	5	3.6	133	96.4
Vaginal Discharge	Yes	7	17.5	33	82.5
	No	10	8.3	110	91.7
Parity	≤2	6	12.8	41	87.2
	>2	11	9.7	102	90.3
Contraceptive Use	Yes	3	6.8	41	93.2
	No	14	12.1	102	87.9

For primers GP5/GP6, the total 25µL PCR reaction mixture contained 5 µL sample, 12.5 µL ready to use PCR mixture (Super Hot Master mix 2x, Bioron GmbH) and 0.4 pmol of each primer. The PCR thermal profile was: 95ºC for 5 minutes, 40 cycles of 94ºC for 30 s, 45ºC for 30s, 72ºC for 30 s and final extension of 5 minutes at 72ºC.

Polymerase chain reaction conditions for TS16 and 18 primers were the same as for the GP primers, except that for TS16 and 18, the annealing temperatures were 61ºC and 63ºC respectively.

For PC03/PCO4 primers, the total 25µL PCR reaction mixture contained 5 µL sample, 12.5 µL ready to use PCR mixture (Super Hot Master mix 2x, Bioron GmbH) and 0.4 pmol of each primer. The PCR thermal profile was 94ºC for 30 s, 51ºC for 30 s, 72ºC for 30 s and 5 minutes final extension at 72ºC.

Amplified PCR products were run on 2% agarose gel and stained with ethidium bromide. The PCR products were identified on the basis of their predicted fragment size.

### Data analysis done by using Statistical Package for Social Sciences version 21 (SPSS 21)

The demographic and clinical variables in both the groups were compared using Chi square test at significance level P value < 0.05 and odd ratio at confidence interval of 95% calculated.

## RESULTS

**Fig.1:**
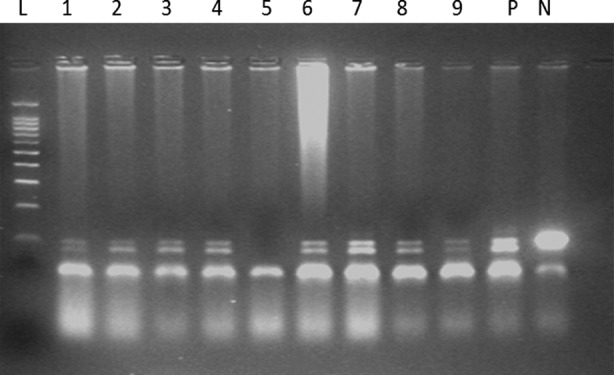
Polymerase chain reaction amplification of human papillomavirus (HPV) type16 in Cervical scrape, Calposcopic /biopsy samples. The products were electophoresed on 2% agarose gel and stained with ethidium bromide. Lane N, negative control; lane P, positive control; lanes 1-9, HPV 16 positive samples; Lane L, molecular size marker (50 base pairs ladder marker).

All the participants were screened through Pap smear for HPV. According to the Pap smear test result, out of 160 healthy women 17 (10.62%) were found Pap smear positive. HPV DNA test on PCR reported that out of them 6 was HSIL (CIN2) which showed high risk of development of cancer. Out of these 5 cases were found Genotype 16 positive whereas no cases were detected for genotype 18. However one case remains undetected repeated twice. The remaining 11 positive cases for Pap smear were LSIL (CIN 1). Out of these, 3 were HPV positive at Genotype 16 whereas 5 were found HPV negative at DNA level and they were advised for follow up. The rest of 3 cases did not join the study after found positive for Pap smear (Fig.1).

### Base line information of healthy women

**Fig.2:**
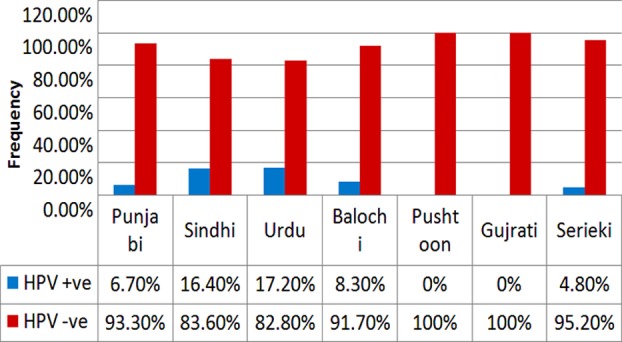
Distribution of Ethnicity.

Overall 160 healthy women selected for screening of HPV, 58 were ≤ 40 years whereas 102 (63.7%) were >40 years of age. Majority of the women (139 i.e. 86.9%) were all single only wife of their husband. For remaining 21 women (13.1%), their husbands were married with more than one wife. Out of 160 healthy women, majority 122 (76.25%) were literate, belonged to middle income group (107 i.e.66.87%), residing in urban area (113 i.e. 70.62%) and having more than 2 children (113 i.e. 70.62%). Only 44 participated women (i.e. 27.5%) were using hormonal contraceptives. Majority of women were Urdu speaking (17.20%) (Fig 2).

### Clinical findings of healthy women screened for HPV

Pap smear +ve result were found more in > 40 years of age group women which have an odd ratio of 2.15 as compared to the females of ≤40 years of age. According to the results, positive Pap smear was found more in those women whose age at first sexual intercourse was > 20 years having an odd ratio of 3.01. In 17 positive pap smear women, history of STD (sexual transmitted disease) was found in 16 cases having an odd 47.55 with 95% CI =6.09-371.36. Previous screening with Pap smear test was done in 17 patients with negative results but repeat screening revealed 3 patients with positive results showing 1.9 times more chances to be screen positive for treatment of pre cancer in women who have not get regular screening for Pap smear.

## DISCUSSION

Our study showed a high incidence of HPV infection in precancerous lesions. We further tried to determine the association of different socio-demographic factors with HPV infection. According to one study, the mean age of participants was 39±4.9 years.^13^ In other studies, the mean age was 38.1±11.04 and 37.5±9.4 years respectively.^14^,^15^ In our study mean age 42.37±5.69 years as compared to above studies.

In various studies majority participants belong to middle social class and were literate but with low education level.^16^ In another study it was assessed that among mediators affecting the progress from HSIL to cancer, education level also played the main role in easing the progression (OR = 4.20, P = 0.066).^17^ In a study 90.6% patients belonged to poor socioeconomic status.^15^ Our study results are similar to this as 72.6% were literate but their literacy level was not determined in our study. Similarly all the participants belonged to low or middle income groups.

In another study high prevalence of abnormal Pap smear was related to, early age at marriage and high parity.^16^ In other studies, parity, tobacco smoking and oral contraceptive use were the main risk factors.^14^,^15^ In our study, out of 17 Pap smear positive cases 10 belonged to that group in which women have got their first sexual inter course in the age of >20 years. However in percentage vise, Pap smear was found more +ve in those women whose age at first sexual intercourse was ≤20 years having an odd of 3.01, parity was >2 children 70.62% and contraceptive use was 50%. One of the studies concluded that oral contraceptive use is not associated with HPV prevalence. Our study results were also similar to it and we have also not found any correlation between the Pap smear positive and use of contraceptive in asymptomatic patients.

In a study prevalence of abnormal Pap smears was 12.2% and the highest prevalence of Pap abnormalities was found in the 41-50 years age group in both populations.^16^ In our study Pap smear was positive in only (10.62%) in healthy women. It is somewhat less than that quoted by other studies but it may be due to less number of participants in our study.

In a study conducted in Karachi by S. Khan HPV was positive in healthy asymptomatic females 15.42% (54/360).^12^ In our study 17/160 were pap smear positive 3 refused, 5 HPV negative and 9 (64.2%) were HPV-DNA positive with abnormal cytology result HSIL,6/9 (66.6%) LSIL, 3/9 (33.3%). This result concluded the proportion of High risk HPV higher in women with abnormal cytology. HPV genotype 16 was the most commonly found genotype in women with (HSIL CIN-2) similarly even in (LSIL-CIN1) HPV genotype 16 was the commonest genotype.

## CONCLUSION

A high incidence of HPV infection found in women with pre cancerous lesion of cervix in Pakistani women.

## RECOMMENDATIONS

Pakistan has a significant burden of carcinoma cervix with rising changing trends of risk factors. It is established that well-organized free camps and regular cervical screening for early diagnosis and good quality cytology can reduce cervical cancer mortality. The introduction of HPV vaccination at age 15 to 25 could also effectively reduce the burden of cervical cancer in the coming decade. The high rate of clinical presentation of patients in the advanced stages can be attributed to the lack of public awareness regarding cervical cancer screening programs. In addition to Pap smear, screening of women once or twice during their lifetime using a high sensitivity test such as HPV genotyping would be more logical and effective in a low resource country. Vaccination against HPV may have a greatest value in developing countries where 80% of global burden of cervical cancer occurs each year and where pap smear screening programs have been largely ineffective.
